# Life-Threatening Hemoptysis From Aorto-Bronchial Fistula in a Patient With Remote History of Aortic Coarctation Repair

**DOI:** 10.7759/cureus.53048

**Published:** 2024-01-27

**Authors:** Tayla E Greene, Sheri P Walls, Bryan A Blakeney, Ademayowa O Ademiluyi, Konstantin G Nestoiter

**Affiliations:** 1 Internal Medicine, Piedmont Athens Regional Medical Center, Athens, USA

**Keywords:** aortic pseudoaneurysm, thoracic aortic coarctation, hemoptysis, life-threatening hemoptysis, large-volume hemoptysis, aorto-bronchial fistula

## Abstract

"Hemoptysis" refers to the expectoration of blood from the respiratory tract. However, "life-threatening hemoptysis" includes any volume that leads to features such as airway obstruction, hypoxia, or hemodynamic instability. We present a case of life-threatening hemoptysis in a 65-year-old male with a history of benign prostatic hyperplasia and uncontrolled hypertension. Radiological investigations revealed a pseudo-aneurysm at the site of a prior thoracic aortic coarctation repair more than 50 years prior in childhood. He required vascular surgical intervention, during which there was evidence of an aorta-bronchial fistula as the likely cause of bleeding. Following the repair and optimal blood pressure control, the patient had no further episodes of hemoptysis and was discharged from the hospital. His case not only adds to the growing body of medical literature reporting hemoptysis as a complication of coarctation repair but also highlights the aorto-bronchial fistula as a possible and potentially catastrophic mechanism for bleeding in these patients.

## Introduction

The precise volume threshold for life-threatening hemoptysis remains controversial, however, when it results in airway obstruction, abnormal gas exchange, or hemodynamic instability, it usually refers to 150 mL of blood expectorated in 24 hours or a bleeding rate >/100 mL/hour [1]. That being said, most cases of hemoptysis are non-life threatening. A 2006 cohort study in Paris of 1087 individuals identified the most common causes of major hemoptysis as bronchitis (20%), cryptogenic (18%), cancer (17%), active tuberculosis (12%), and tuberculosis sequelae (13%) [2]. A report in 2018 of 606 patients with hemoptysis, showed malignancy (19%), pneumonia/lung abscess (19%), and bronchiectasis (15%) as the most common causes [3]. The cause of hemoptysis in our patient is exceedingly rare. Fistula formation between blood vessels and the tracheo-bronchial tree can occur in the setting of vascular inflammation which can arise following surgery such as stent placement, infection, or an indwelling vascular device.

## Case presentation

A 65-year-old male with a history of benign prostatic hyperplasia, uncontrolled hypertension, and thoracic aortic coarctation repair 54 years prior, presented with life-threatening hemoptysis. It occurred suddenly and in bouts of over 100 ml of bright red blood each time. When emergency medical services (EMS) arrived, he was hypotensive, diaphoretic, and hypoxic with oxygen saturation as low as 80% and was placed on supplemental oxygen via nasal cannula. He had a brief episode of syncope and was resuscitated with intravenous fluid. By the time he arrived at the emergency department, the hemoptysis had ceased and his vitals were hemodynamically stable. His initial laboratory investigations are reported in Table 1.

**Table 1:** Initial laboratory investigations

Blood Investigation	Result	Reference Range
White blood cell (10^3^/µL)	8.1	4 – 10.50
Hemoglobin (g/dL)	14.6	13.8 – 17.2
Platelets (10^3^/µL)	274	130 – 400
Blood urea nitrogen mg/dL	15	6 – 20
Creatinine (mg/dL)	0.99	0.50 – 1.20
Erythrocyte sedimentation rate (mm/HR)	8	0 – 30
C reactive protein (mg/dL)	0.07	0.02 – 1.00
Procalcitonin (ng/mL)	0.02	0.00 – 0.50

**Figure 1:**
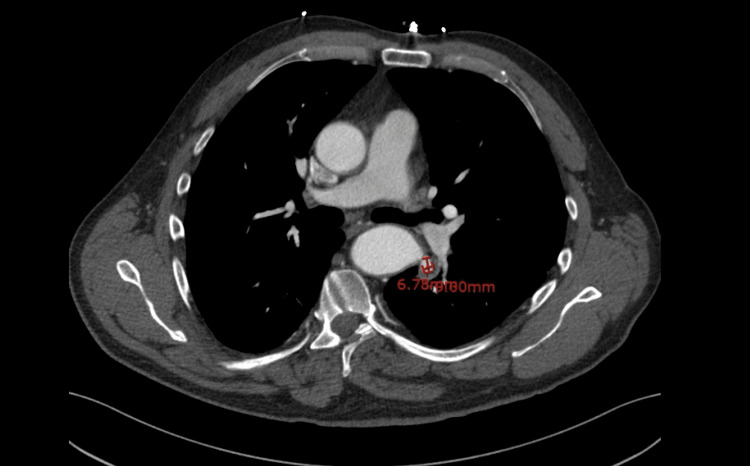
CT angiogram of thoracic aorta showing 10 x 9 mm pseudoaneurysm projecting laterally from the thoracic aorta at the level of previous coarctation repair

The patient's chest X-ray was unremarkable, however, a subsequent Computed Tomography (CT) Chest Angiogram performed was negative for pulmonary embolism but showed bilateral patchy ground-glass opacities. A bronchoscopy was considered, however, due to his known history of repair of thoracic coarctation of the aorta, a CT angiogram of the thoracic aorta was pursued first, which showed a 10 x 9 mm pseudo-aneurysm at the level of the previous repair (Figure 1).

Vascular surgery was consulted, and the patient underwent a Thoracic Endovascular Aortic Repair (TEVAR) of the pseudoaneurysm. Intra-operatively, an aorto-bronchial fistula was identified as the cause of the hemoptysis. 

## Discussion

Hemoptysis as the presenting symptom of a pseudo-aneurysm or aorta-bronchial fistula is rare, but there are now several reports in the literature of its occurrence post-aortic coarctation repair. It represents an often misdiagnosed entity with a likely underestimated incidence and more than 30% of cases are diagnosed at autopsy [4].

Particularly unique about this presentation, however, is the timeline. Most of the reported cases of significant hemoptysis from aorto-bronchial fistula have occurred within much shorter intervals following the initial surgery. For example, Kansal and Nagpal described a 46-year-old patient with hemoptysis who had coarctation repair at the age of 17 [5]. A patient deems a procedure from over 50 years ago irrelevant and does not mention it, or they may not remember it at all. This further enforces the importance of thorough and intentional history-taking, especially as the adult population with a remote history of invasive vascular and other surgical procedures grows [6].

Hemoptysis from aorto-bronchial fistulas may range from small herald bleeds to large volume bleeding with exsanguination, so left untreated this could have led to catastrophic outcomes [7]. It was felt that his significantly uncontrolled blood pressure over time may have also contributed to the development of pseudo-aneurysm and fistula formation. While the initial CT imaging done to rule out pulmonary embolism showed bilateral ground-glass opacities prompting further evaluation, a thorough history and high index of suspicion initially could have led to CT thoracic angiogram as the initial imaging study of choice or both being performed at the same time. The CT angiogram provided a detailed view of the aneurysm and this led to definitive management.

## Conclusions

Even with advances in vascular surgical techniques, complications of invasive procedures are inevitable. Pseudo-aneurysms and aorta-bronchial fistulas are rare but potentially catastrophic complications if left untreated. They have a place on the differential list for any patient with a history of vascular intervention who presents with hemoptysis, regardless of severity. It is possible, regardless of how long it has been since the surgery. Thorough history-taking is essential, and a CT angio/aortogram should be considered early in the presentation to delineate anatomy and guide management if the index of suspicion is high.
